# Delpinium uncinatum mediated green synthesis of AgNPs and its antioxidant, enzyme inhibitory, cytotoxic and antimicrobial potentials

**DOI:** 10.1371/journal.pone.0280553

**Published:** 2023-04-04

**Authors:** Hina Rehman, Waqar Ali, Mohammad Ali, Nadir Zaman Khan, Muhammad Aasim, Ayaz Ali Khan, Tariq Khan, Muhammad Ali, Ashaq Ali, Muhammad Ayaz, Muzamil Shah, Syed Salman Hashmi

**Affiliations:** 1 Department of Biotechnology, University of Malakand, Chakdara, Lower Dir, Pakistan; 2 Centre for Biotechnology and Microbiology, University of Swat, Swat, Pakistan; 3 Department of Biotechnology, Quaid-i-Azam University, Islamabad, Pakistan; 4 Center for Excellence in Science and Applied Technology, Islamabad, Pakistan; 5 Department of Pharmacy, University of Malakand, Chakdara, Lower Dir, Pakistan; King Abdulaziz University, SAUDI ARABIA

## Abstract

Green synthesis of nanoparticles is becoming a method of choice for biological research due to its environmentally benign outcomes, stability and ease of synthesis. In this study, silver nanoparticles (AgNPs) were synthesized using stem (S-AgNPs), root (R-AgNPs) and mixture of stem and root (RS-AgNPs) of *Delphinium uncinatum*. The synthesized nanoparticles were characterized by standardized techniques and evaluated for their antioxidant, enzyme inhibition, cytotoxic and antimicrobial potentials. The AgNPs exhibited efficient antioxidant activities and considerable enzyme inhibition potential against alpha amylase, acetylcholinesterase (AChE) and butyrylcholinesterase (BChE) enzymes. S-AgNPs showed strong cytotoxicity against human hepato-cellular carcinoma cells (HepG2) and high enzyme inhibitory effect (IC50 values 27.5μg/ml for AChE and 22.60 μg/ml for BChE) compared to R-AgNPs and RS-AgNPs. RS-AgNPs showed significant inhibition of Klebsiella pneumoniae and Aspergillus flavus and exhibited higher biocompatibility (<2% hemolysis) in human red blood cells hemolytic assays. The present study showed that biologically synthesized AgNPs using the extract of various parts of D. uncinatum have strong antioxidant and cytotoxic potentials.

## Introduction

Nanobiotechnology has emerged as a flourishing scientific field dedicated to the synthesis of multifunctional nanoparticles (NPs) by using green processes and biological resources [1]. NPs due to their small size (1–100 nm) and unique surface area to volume ratio exhibit interesting physical and biochemical properties [2, 3]. NPs can be synthesized by physical and chemical methods however, there are certain limitations associated with these methods. Synthesis using physical methods is energy consuming and is therefore economically not feasible. Chemical methods can generate toxic and hazardous waste. Alternatively, biological methods are safe, simple, rapid and cost effective [4]. Among the biological resources, enzymes, microorganisms, fungi and whole plants or specific parts of a plant have been used for NPs synthesis [5, 6]. Plants are by far the most widely exploited biological source for the synthesis of NPs. This is due to the fact that plant secondary metabolites actively participate in capping and stabilization of NPs [7]. Additionally, plants are easily available, and cheapest source among biological resources which makes their use in NPs biosynthesis more common [8]. Diverse applications of plant based NPs are reported in different fields like health, food, environment, cosmetics, optics, electronics, space industries, drug and gene delivery, chemical industries, energy, single electron transistors, light emitters and nonlinear optical devices etc. [9].

Noble metal NPs, like silver NPs (AgNPs) are studied extensively due to their multifunctional nature [10, 11]. In ancient times, the use of silver (Ag) was very popular owing to its antimicrobial potential. The modern era therefore exploits the nano form of silver i.e. AgNPs in a number of biomedical applications [12]. Apart from this, AgNPs have also been used for a number of applications including viral inhibition assays, anti-cancer activities, wound dressing, food preservatives and water treatment [13]. AgNPs have also been used extensively as antifungal, antioxidant, anti-inflammatory and anti-angiogenic agents [14]. Green synthesis of AgNPs is frequently reported using medicinal plants. The use of medicinal plants is preferred for biosynthesis of NPs since they have been extensively studied and their biochemical profile is well documented. Moreover, the use of medicinal plants for biosynthesis of NPs eliminates the use of noxious chemicals that are critical for capping and stabilization of NPs [15].

The species of Delphinium are found mostly at the high altitude of the western region of Himalayas (2400 to 3650 m). *D*. *uncinatum* and related species have been used to treat multiple diseases such as fever, sour throat, cough, cold, gout, vomiting, rheumatism, stomach pain, diarrhea and epilepsy [16, 17]. Different bioactive complex structure compounds like alkaloids e.g. uncinatine, 14-acetylperegrine, 14 acetylvirescenine, condelphine, delbrusine [18, 19], flavonoids, delphinin [20], violdelphin [21], cyanodelphin [22] and phenolic compounds e.g. 2,5,6-trihydroxypiperonylic acid and methyl ester [23] have been reported recently from Delphinium species. The present work aimed to synthesize biologically stable AgNPs using different parts of *D*. *uncinatum*, and to evaluate their antioxidant, enzyme inhibition, cytotoxic and antimicrobial potentials. AgNPs ranging in size from 24.6 nm to 32 nm showed excellent potential in all the aforementioned biological assays. The synthesized AgNPs effectively inhibited enzymes like protein kinase, alpha-amylase and cholinesterase suggesting that the NPs have anti-diabetic and anti-inflammatory potential. The antimicrobial potential of the NPs was also comparable to that of standard antibiotic. NPs also proved to effectively scavenge the reactive oxygen species (ROS) and showed effective inhibition of HepG2 cell lines hinting at their cytotoxic nature.

## Materials and methods

**Fig 1:**
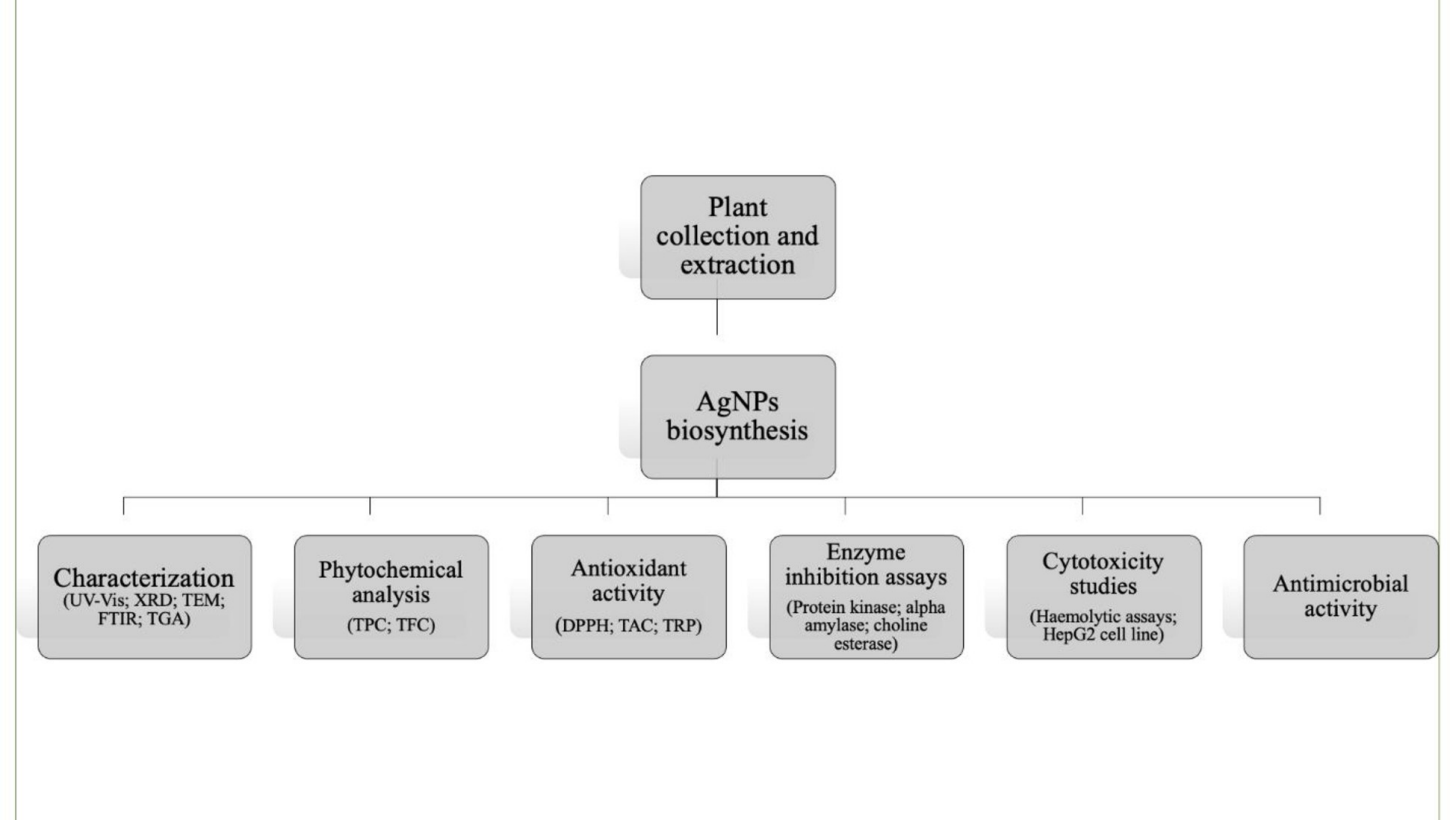
Schematic diagram of experiments performed.

Following are the key materials and methods used in the current research work along with the schematic diagram (Fig 1) provided in this section.

### Preparation of plant material

Fresh plant of *D*. *uncinatum* was collected from District Swat of the Province Khyber Pakhtunkhwa, Pakistan in 2017. The plant was taxonomically verified at the Department of Botany, University of Swat. The plant materials were washed with distilled water and then shade dried. Once the plant material was properly dried, the roots and stem were separated and ground to fine powder. The powdered material was stored in airtight jars for extraction [24].

### Preparation of plant extracts and AgNO_3_ aqueous solution

Aqueous tissue extracts were prepared by boiling dried root powder (0.5 g), stem powder (0.5 g) and a mixture of root and stem powder (0.25 g each) separately in 100 ml distilled water for 5 to 10 minutes. The prepared aqueous extracts were kept at room temperature (25°C) for an hour and then filtered 3 to 5 times using Whatman filter paper and nylon fabric. The stock aqueous extract was diluted several times by 2-fold serial dilution method and all extracts were stored at 4°C to avoid potential contamination. To prepare 10 mM stock solution of silver nitrate, 0.0169 g of AgNO3 was dissolved in 100 ml distilled water that was subsequently serially diluted as 8 mM, 6 mM, 4 mM and 2 mM solutions [25].

### Biosynthesis of AgNPs

To synthesize AgNPs, aqueous tissue extracts of roots (R-extract), stem (S-extract) and mixture of root and stem (RS-extract) (5 g/L, 2.5 g/L and 1.25 g/L) were treated with 8 mM, 6 mM, 4 mM, 2 mM or 1 mM of silver nitrate in 1:1 (v/v) ratios. The reaction mixtures were kept at room temperature for 24 hrs and color changes were recorded [26].

### Characterization of the AgNPs

#### UV-vis-spectrophotometric analysis

UV-vis spectra of tissue extract and AgNO^3^ mixture aliquots (1.0 ml) was monitored as a function of time of reaction using UV-vis double beam spectrophotometer (HALO DB-20, Dynamica Scientific Ltd, UK) in 300–600 nm spectral range. Unreacted AgNO^3^ and tissue extracts were removed by washing out and pelleting AgNPs by centrifugation. Briefly, reaction mixtures (40 mL) were centrifuged at 12,000×g in falcon tube (50 ml) for 10 min at room temperature. The supernatants were removed and distilled water was added to each tube containing the pellets in order to make a final volume of 40 mL. The pellets were homogenized in distilled water using hot plate and the process of centrifugation was repeated. The washing cycle was repeated 3 to 5 times. The resulting AgNPs were dried at room temperature for characterization.

#### X-ray Diffraction (XRD) analysis

XRD analysis was used to find out the crystalline nature of the synthesized AgNPs (PAN-analytical X’ Pert PRO Holland) with Cu-kα radiations at a scan rate of 10 s^-1^. The analyzed material was finely ground, and average bulk composition was determined. The particle or grain size of the AgNPs was calculated using Debye Sherrer’s equation (D = Kλ/β cos θ) where, *D* is the average particle size, *k* is the shape factor (0.9), *λ* is the X-ray wavelength (1.5406 A˚), *β* is the full width at half maximum of the peak (FWHM) and *θ* is the diffraction angle.

#### Transmission Electron Microscopic (TEM) analysis

For TEM based analysis, samples were prepared by dissolving 100 mg of AgNPs in 50% ethanol followed by sonication for 25 min. 10 μl of each sample was dropped on parafilm and a grid was placed over it for 5–15 min, followed by TEM measurements (FEI’s Technai™ G2 Transmission Electron Microscope) after 24 hrs.

#### Fourier-transform Infrared Spectroscopy (FT-IR)

FT-IR spectral measurements were carried out for the identification of potential phytochemicals involved in capping and stabilization of AgNPs using spectrophotometer (TENSOR-II Spectrophotometer, Bruker Optics, UK) with frequency range 4000–600 cm^-1^. The instrument was equipped with ATR assembly containing diamond crystal. Recorded spectra were an average of around 120 scans.

#### Thermogravimetric analysis (TGA)

The thermogravimetric characteristics of AgNPs were analyzed using TGA/DSC1, Mettler Toledo, Star e System software. Two clean crucibles made up of alumina were used for sample and reference. 14.7 mg of each sample was kept in crucible and subjected to heat at increasing rate of 5°C min^−1^ from room temperature to 800°C with continuous movement of nitrogen gas at a flow rate of 40 ml min^−1^.

### Preliminary phytochemical analysis

#### Total Phenolic Content (TPC) analysis

Analysis of total phenolic content was carried out according to Folin–Ciocalteu reagent method [27] with minor modifications using microplate reader (Biotek USA, microplate reader Elx 800). 20 μL of each sample (4 mg/mL) was mixed with 90 μL of Folin-Ciocalteu reagent, followed by incubation for 5 min and then addition of 90 μL of sodium carbonate. Absorbance of samples was taken at 630 nm. The results were showed as microgram equivalents of gallic acid per milligram of the sample (μg GAE/mg) [28].

#### Total Flavonoid Content (TFC) analysis

To determine total flavonoid content, Aluminum trichloride (AlCl3) colorimetric method was used with minor alterations for the suitability of system described previously [29]. In a 96-well plate, volume of 20 μL from each sample (4 mg/mL) was shifted in a respective well, followed by addition of 1 μL of aluminum chloride (10%), 10 μL of potassium acetate (1M) and 160 μL of distilled water. The resulting mixture was kept for 30 min at normal room condition. After some time, with the help of a microplate reader, the absorbance of samples was measured at a 630 nm. Standard sample of DMSO and quercetin was used as a negative and positive control respectively. The results were showed as microgram equivalents of quercetin per milligram of the sample (μg QE/mg).

### Antioxidant studies

#### DPPH anti radicals assay

Antioxidant activity of the synthesized NPs (200–1 μg/mL) was determined in terms of in vitro scavenging potential of samples for the free radical 2,2-diphenyl 1-picrylhydrazyl (DPPH) [30]. Briefly, 2.4 mg of DPPH was added 25 ml methanol to prepare reagent solution. Ascorbic acid and DMSO were taken as positive and negative controls respectively. Subsequently, 20 μL of each test sample was mixed with 180 μL of reagent solution to prepare reaction mixtures. After a resting phase of an hour, each mixture was analyzed at 517 nm using spectrophotometer. The percent DPPH scavenging was calculated as % Scavenging = 1−SAB/CAB ×100, where SAB means absorbance of sample and CAB means absorbance of control respectively.

#### Total Antioxidant Capacity (TAC) assay

Total antioxidant capacity was determined by phosphomolybdenum method as described previously [31]. Briefly, 20 μL of each test sample was mixed with 180 μL of reagent mixture (0.6 M H_2_SO_4_, 28 mM NaH_2_PO_4_, 4 mM (NH_4_) 6Mo_7_O_24_.4H_2_O). Reading of samples was taken at 695 nm after a resting phase of 90 min at 95°C. The results were showed as microgram equivalents of ascorbic acid per milligram of the sample (μg AAE/mg).

#### Total Reducing Power (TRP) assay

Total reducing power of the synthesized NPs was evaluated using potassium ferricyanide, as described previously [32]. 40 μL of each test sample was mixed with 50 μL of phosphate-buffered saline and the mixtures were incubated at 50°C for 20 min, followed by addition of 50 μL of trichloro acetic acid (10%) and centrifugation at 3000 rpm for 10 min. The collected supernatant (166.6 μL) was mixed with 33.3 μL of FeCl_3_ (0.1%). The absorbance readings were taken at 630 nm and the results were presented as ascorbic acid equivalents per milligram.

### Enzyme inhibitory studies

#### Protein kinase inhibition assay

To evaluate protein kinase inhibition potential of synthesized AgNPs, Streptomyces 85E strain cultured in ISP4 minimal media was treated with synthesized AgNPs samples. After making a uniform bacterial lawn, sterile filter paper discs were placed on culture plates and loaded with 6 μL of AgNPs (4 mg/ml to 0.5 mg/ml; 2-fold dilutions). Surfactin was used as positive control, Cultures were incubated at 30°C for 72 h and zones of inhibition were measured in mm [33].

#### Alpha amylase inhibition assay

To study the alpha amylase (α-amylase) enzyme inhibition potential of synthesized AgNPs, microplate method was used with slight modification [34]. The reaction mixture containing 15 μl phosphate buffer (pH 6.8), 25 μl α-amylase enzyme (Uni-Chem Chemical Reagents) (0.14 U/ml), 10 μl sample (4 mg/ml DMSO) and 40 μl (2 mg/ml in potassium phosphate buffer) starch solution was incubated at 50°C for 30 min in 96-well plate, followed by addition of 20 μl of 1M HCl. About 90 μl of iodine reagent (5 mM iodine, 5 mM potassium iodide in phosphate buffer) was added to each well. DMSO and acarbose (250 μM) were used as negative and positive control respectively. Absorbance of samples was measured at 540 nm. The percent α-amylase inhibition was calculated as:

% α-amylase inhibition = (SABS−NABS)/ (BABS−An) ×100

Where NABS = Absorbance of negative control, SABS = Absorbance of sample and BABS = Absorbance of blank.

#### Cholinesterase (AChE/BChE) inhibition assay

Slight modified Elman’s methodology [35] was used to investigate the inhibitory potency of AgNPs against acetyl-cholinesterase (AChE) and butyryl-cholinesterase (BChE). Enzymes including acetylcholinesterase (AChE), source Electrophorus electricus (CAS number 9000-81-1), purchased from Sigma Aldrich, St. Loius, MO, United States and Butyrylcholinesterase (BChE), source equine serum (CAS number: 9001-08-5), Sigma Aldrich GmbH, Germany were used in the study. To performed the assay, the synthesized AgNPs were dispersed in PBS and other substrate solutions like DTNB (5,5-dithiobisnitrobenzoic acid), butyrylcholine iodide and acetylcholine iodide were prepared in distilled water and kept at 8°C. The final concentrations of enzymes were 0.03 U/mL for AChE and 0.01 U/mL for BChE. Galantamine hydrobromide which was prepared in methanol at the concentration of 10 mg/mL was used as a positive control, while the reaction mixture without test sample was used as a negative control. Afterward, additional DTNB complexes exhibited yellow color which were further used to record its absorbance at 412 nm using UV-VIS spectrophotometer. The three biogenic AgNPs and galantamine percent enzyme activity and percent enzyme inhibition were calculated with change in absorption rate with time. V = ΔAbs/Δt; Percent enzyme activity = V/Vmax × 100; and % inhibition = 100—percent enzyme activity.

### Cytotoxicity studies

#### Human Red Blood Cells (hRBCs) compatibility test

Human red blood cells (hRBCs) hemolytic assay was carried out to study the compatibility of synthesized AgNPs using fresh isolated RBCs [36]. The blood was obtained from healthy donors only for testing the haemolytic potential of NPs. A written consent was obtained from the donors. The study was approved by the Advanced Studies and Research Board (ASRB) of the University of Malakand in its 59^th^ meeting. 5.0 ml of the fresh blood, collected from healthy individuals, was centrifuged (14,000 rpm for 5 min) to isolate erythrocytes. The suspension of erythrocytes was prepared by adding phosphate buffered saline (pH 7.2) to of isolated erythrocytes (200 μL). About 100 μL of different concentrations of AgNPs was added to 100 μL of erythrocytes suspension and incubated for 1.0 h at 35°C in 96-well plate. After incubation, the samples were centrifuged (10,000 rpm for 10 min). To find out the release of total hemoglobin from the collected supernatant, the absorbance was monitored at 530 nm. DMSO was used as negative and Triton X-100 was used as a positive control. The following equation was used to found out the percent hemolysis.

% Hemolysis = 100 ×((ABs -ABNC)/(ABPC-ABNC))

Where, “Abs” absorbance of supernatant, “AB NC” of negative control and “ABPC” absorbance of positive control.

#### HepG2 cell line cytotoxicity assay

Human hepatocellular carcinoma cells (ATCC HB-8065) were cultured in Dulbecco’s Modified Eagle’s medium having Fetal calf serum (10%), supplemented with 1.0 mM Na-pyruvate, 2.0 mM L-glutamine, 100 μg/mL streptomycin (37°C) and 100 U/mL penicillin in a 5% humidified CO2 atmosphere. Harvesting of the cells was done with trypsin/EDTA (0.5 mM) for 1.0 min at room temperature. The synthesized AgNPs were assessed by sulforhodamine B (SRB) assay for their cytotoxic activity against HepG2 cell line as originally described [37]. AgNPs (20 mg) were suspended in distilled water (1.0 ml) for cytotoxicity screening. Cancer cell line (HepG2) having confluence rate greater than 90% were incubated at a density of 12000 cells/well in a 96-well plate and allowed to stick for 24 h at 37°C. Afterwards, these sticky cells were treated with NPs (200 μg/ml) for 24 h and fixed with the help of pre-chilled trichloroacetic acid (50%) and further incubated for 1 h at 4°C, followed by three time washing with deionized water. The produced plates were then dried with air and stained with dye sulforhodamine B (0.01%), followed by incubation (30 min, at room temperature). For the removal of unbounded dye, acetic acid (1%) was used. Solubilization of the SRB dye was done by the addition of 100 μl of Tris (10 mM) having pH 8 into each well for 5 min at room temperature. DMSO and doxorubicin (34 μM) were used as a negative and positive control respectively. Blanks describing the optical density of the background consisted of only the sample and controls of the media. Snapshots of the results were captured using Olympus light microscope (CK2) equipped with digital camera. Absorbance of the samples was evaluated using Microplate reader (Platos R 496, AMP) at wavelength 565 nm. Experimentation was repeated two times with triplicates for every test sample. Cell viability and inhibition percentage comparative to the untreated sample was calculated using the following formulas: % Cells viability = 100 ×((ABS -ABC)/(ABUC-ABM)) and % cell inhibition = 100 –Cell viability (%).

Whereas, absorbance patterns of the sample (Abs), Control (AbC), Untreated Cells (AbUC) and Media (AbM) have been mentioned above.

### Antimicrobial studies

The antibacterial and antifungal assays of synthesized AgNPs were performed against four bacterial strains [*Staphylococcus epidermidis* (ATCC 14990), *Bacillus subtilis* (ATCC 6633), *Pseudomonas aeruginosa* (ATCC 9721), and *Klebsiella pneumoniae* (ATCC 4617)] and three fungal strains [*Fusarium solani* (FCBP 0300), *Aspergillus flavus* (FCBP 0064), and *Aspergillus fumigates* (FCBP 66)] using agar well diffusion method [38, 39]. One day old inoculated microbial inoculums (OD 0.5) were spread on the entire surface of agar plate with the help of a sterile cotton bud and holes were punched (6 mm) using a sterile hole borer. About 20 μL of AgNPs of different concentrations (1.0 mg/ml, 2.0 mg/ml, 4.0 mg/ml, 5.0 mg/ml and 10 mg/ml) were introduced into specific labeled wells with positive control (penicillin for bacterial and amphotericin b for fungal culture) and negative control (DMSO). The prepared plates were then placed in an incubator for 24 h in case of bacterial and 48 h in case of fungal strains. The growth inhibition zones were measured with the help of Vernier caliper in mm.

### Statistical analysis

Biological synthesis, phytochemical analysis and biological activities of each sample were performed in triplicate and the results were presented as mean or mean ± SD by using the software GraphPad Prism. All experiments were repeated at least twice. Graphs were generated using the software Origin 8.5.

## Results and discussion

### Total phenolic and flavonoid contents

Phenolic compounds are antioxidant agents which act as a terminators of free radicals. In this study, the total phenolic contents of the given samples at concentration of 4 mg/ml were recorded. AgNPs synthesized using RS-extract (RS-AgNPs) showed higher phenolic content (70 ± 0.3 μg GAE/mg) as compared to AgNPs synthesized using R-extract alone (R-AgNPs) (65 ± 0.8 μg GAE/mg) and AgNPs synthesized using S-extract alone (S-AgNPs) (23 ± 0.08 μg GAE/mg). Similarly, maximum total flavonoid contents was recorded for RS-AgNPs (22 ± 0.5 μg QE/mg) followed by R-AgNPs and S-AgNPs (Table 1).

**Table 1:** Results of antioxidant and phytochemical studies.

	Secondary metabolites		Antioxidant activities		
Sample	TFC	TPC	DPPH	TAC	TRP
	(μg QE/mg)	(μg GAE/mg)	(% Scavenging)	(μg AAE/mg)	(μg AAE/mg)
R-AgNPs	21± 1.3	56± 0.8	71±1.3	120±1.1	22±1.2
S-AgNPs	3± 0.3	23± 0.08	77±1.5	61±1.1	24±0.64
RS-AgNPs	22± 0.5	70± 0.3	69±1.4	122±0.69	33±1.2

### UV-Vis spectrophotometry

**Fig 2:**
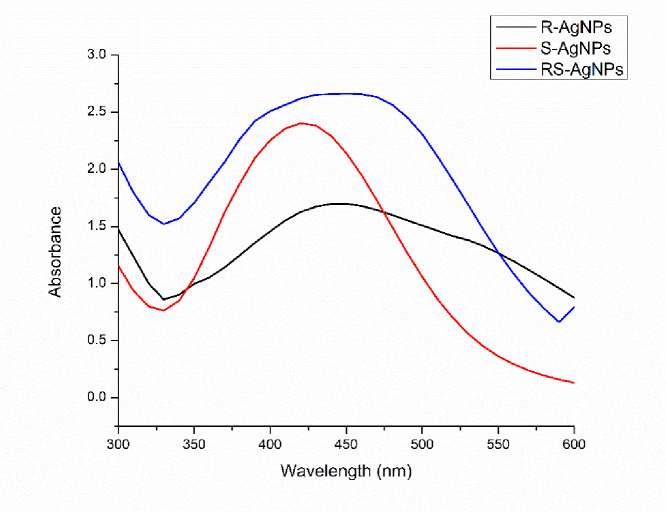
UV-Vis spectrophotometric analysis of AgNPs synthesized using various parts of *D*. *uncinatum*.

Recently, the synthesis of NPs from medicinal plants dry extracts and heterocyclic compounds are center of contemplation owing to the existence of plethora of phytochemicals, followed by environment friendly nature and easier synthesis protocols [40]. In our study, aqueous extracts of different tissues of the medicinal plant *D*. *uncinatum* (R-extract, S-extract and RS-extract) were mixed with different concentrations of AgNO3 for AgNPs synthesis. Following mixing, the appearance of dark brown color of mixtures after 24 h indicated the synthesis AgNPs. The intensity of brown color to dark brown increased with time. Previously, different researchers have reported the duration of the synthesis of AgNPs using different plant tissues [41]. The narrow surface plasmon resonance (SPR) of synthesized AgNPs presented peaks from 300–600 nm regions in which the highest absorbance was recorded for RS-AgNPs i.e. 450 nm. Similarly, R-AgNPs exhibited maximum absorbance at 450 nm whereas S-AgNPs exhibited maximum absorbance at 430 nm (Fig 2). Since AgNPs exhibit characteristic peaks between 400–480 nm, our results confirm the reduction of silver nitrate into AgNPs.

### XRD analysis

**Fig 3:**
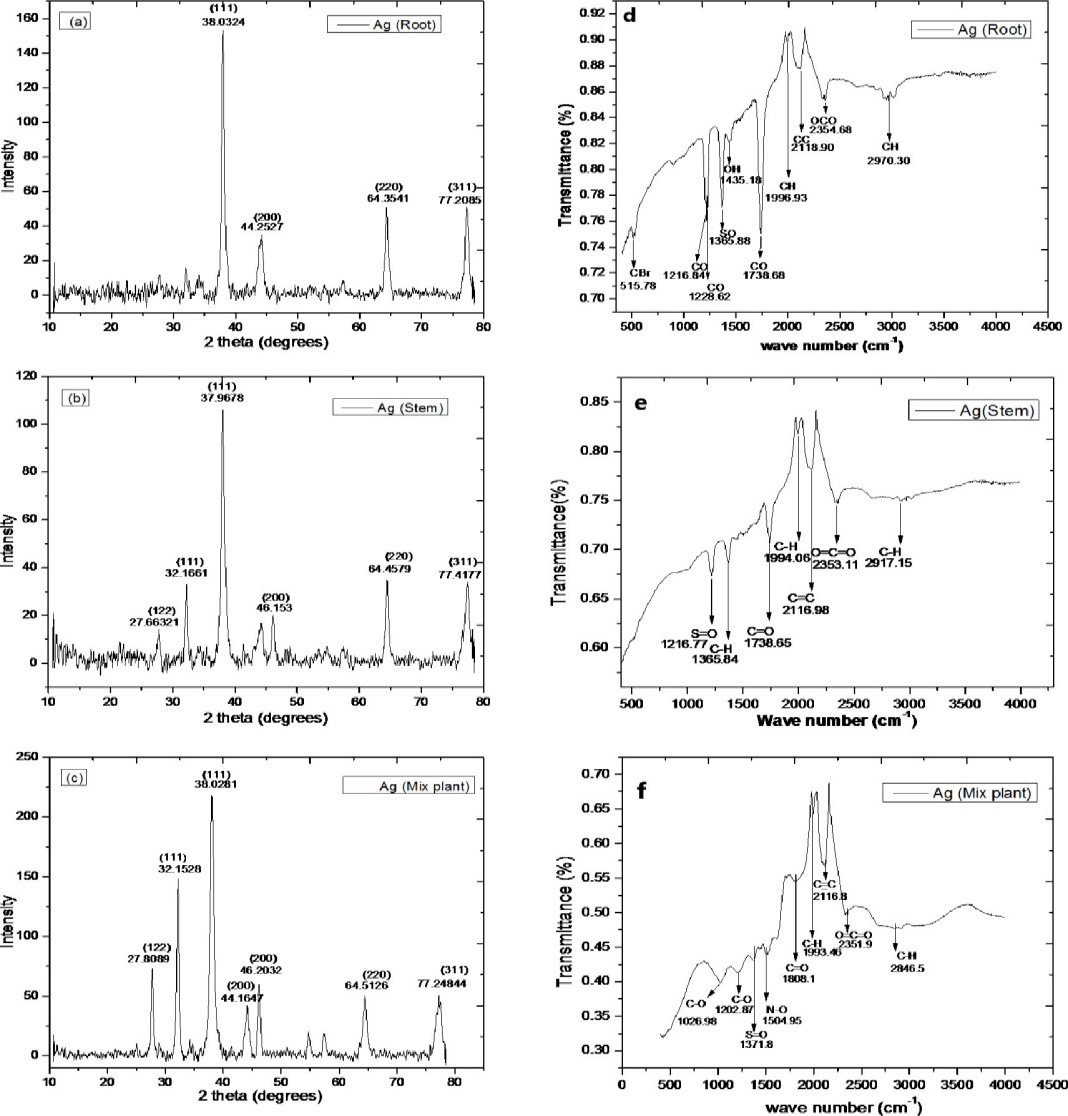
X-ray diffraction (XRD) (**a-c**) and fourier transform infrared spectroscopy (FTIR) (**d-f**) profiles of the R-AgNPs, S-AgNPs and RS-AgNPs respectively.

XRD is a powerful tool for phase identification, determination of crystallinity and information on unit cell dimensions. The XRD spectrum analysis is mainly used to check the NPs crystalline nature. Fig 2 clearly indicates that the green synthesized AgNPs were crystalline in nature. The Bragg reflections of AgNPs for all the samples were observed at 2*θ* values separately. For R-AgNPs, four peaks were recorded at 38.03°, 44.25°, 64.35°, and 77.20° in the experimental diffractogram. These correspond to (hkl) values (111), (200), (220) and (311) lattice planes. The diffractogram of S-AgNPs showed six different peaks at 27.71°, 32.16°, 37.96°, 46.15°, 64.45° and 77.41°. The observed values correlate to the corresponding planes of crystal lattice i.e. (122), (111), (111), (200), (220) and (311) of the powder diffraction Standards (JCPDS: 89–3722) file. Similarly, the RS-AgNPs diffractogram indicated different peaks at 27.80°, 32.15°, 38.02°, 44.16°, 46.20° and 64.51° which corresponds to (122), (111), (111), (200), (200) and (220). All the noted values that the synthesized NPs have face centered cubic structure. The average sizes of the R-AgNPs, S-AgNPs and RS-AgNPs calculated using the Debye Scherrer equation was around 15 nm, 14 nm and 13 nm respectively (Fig 3A–3C). The results of the previous published work are in agreement with our experimental results [41, 42].

### FTIR analysis

FTIR spectrum of the AgNPs synthesized from three different parts of D. uncinatum showed a variety of functional groups attached to the synthesized NPs (Fig 3D–3F). The overall spectra of R-AgNPs exhibited 10 absorption peaks in the range of 500–4000 cm^-1^ (Fig 3D–3F). A peak at 2970.3 cm^-1^corresponds to the stretching vibrations of C-H bond of alkane group. The peak at 2354.6 cm^-1^ was observed due to the O = C = O stretching. The peak at 2118.9 cm^-1^can be attributed to stretching vibrations of C≡C linkage in alkynes. Peak at 1996.9 cm^-1^ indicated the stretching vibrations of C-H in aromatic compounds. Similarly, the peak at 1738.6 cm^-1^ could be assigned to C = O stretching in aldehydes. The band at peak 1435.1 cm^-1^ can be attribute to O-H bending vibration of carboxylic acid. The absorption peak at 1365.8 cm^-1^accounts for S = O stretching bond of sulfonamide. The absorption bands at 1228.62 cm^-1^ 1216.8 cm^-1^ and 515.78 cm^-1^ were observed indicating the stretching of C-O bond of vinyl ether, ester and C-Br functional group of the halo compounds. In case of S-AgNPs, the FTIR spectra revealed a total of seven peaks between 500–4000 cm^-1^ (Fig 3E). The peak at 2917.15 cm^-1^ indicated C-H stretching of alkanes. The peak at 2353.11 cm^-1^ indicates O = C = O stretching vibrations. The peaks at 2116.98 cm^-1^ and 1994.06 cm^-1^ can be assigned to stretching and bending of functional groups of C≡C and C-H of the corresponding alkyne and aromatic compounds. Similarly, a combination of the strong stretching and medium bending bonds were observed at peaks 1738.65 cm^-1^, 1365.84 cm^-1^ and 1216.77 cm^-1^ indicating C = O, C-H and S = O functional groups of aldehyde, alkane and sulfonyl chloride. RS-AgNPs exhibited strong, medium and weak peaks between 500–4000 cm^-1^ (Fig 3F). The medium and strong bands observed at 2846.51 cm^-1^, 2351.91 cm^-1^ represents the C-H and O = C = O stretching of alkane and carbon dioxide. Weak peaks noted at 2116.82 cm^-1^ and 1993.46 cm^-1^ denoted the stretching C≡C and C-H functional groups of the alkyne and aromatic compound. Strong absorption bands at 1808.09 cm^-1^, 1504.95 cm^-1^, 1371.8 cm^-1^, 1202.87 cm^-1^ and 1026.98 cm^-1^ exemplified the C = O, N-O, S = O, C-O and C-O stretching of acid halide, nitro compound, sulfonate, alkyl aryl ether and vinyl ether respectively. The overall results of FTIR study of the synthesized NPs revealed that plant phytochemicals were actively involved in the process of reduction, capping and stabilization of AgNPs. Phytochemicals with hydroxyl (-OH), carboxyl (-C = O) and alkane (C-H) as a functional group were mainly involved in the reduction and stabilization of AgNPs.

### TEM analysis

**Fig 4:**
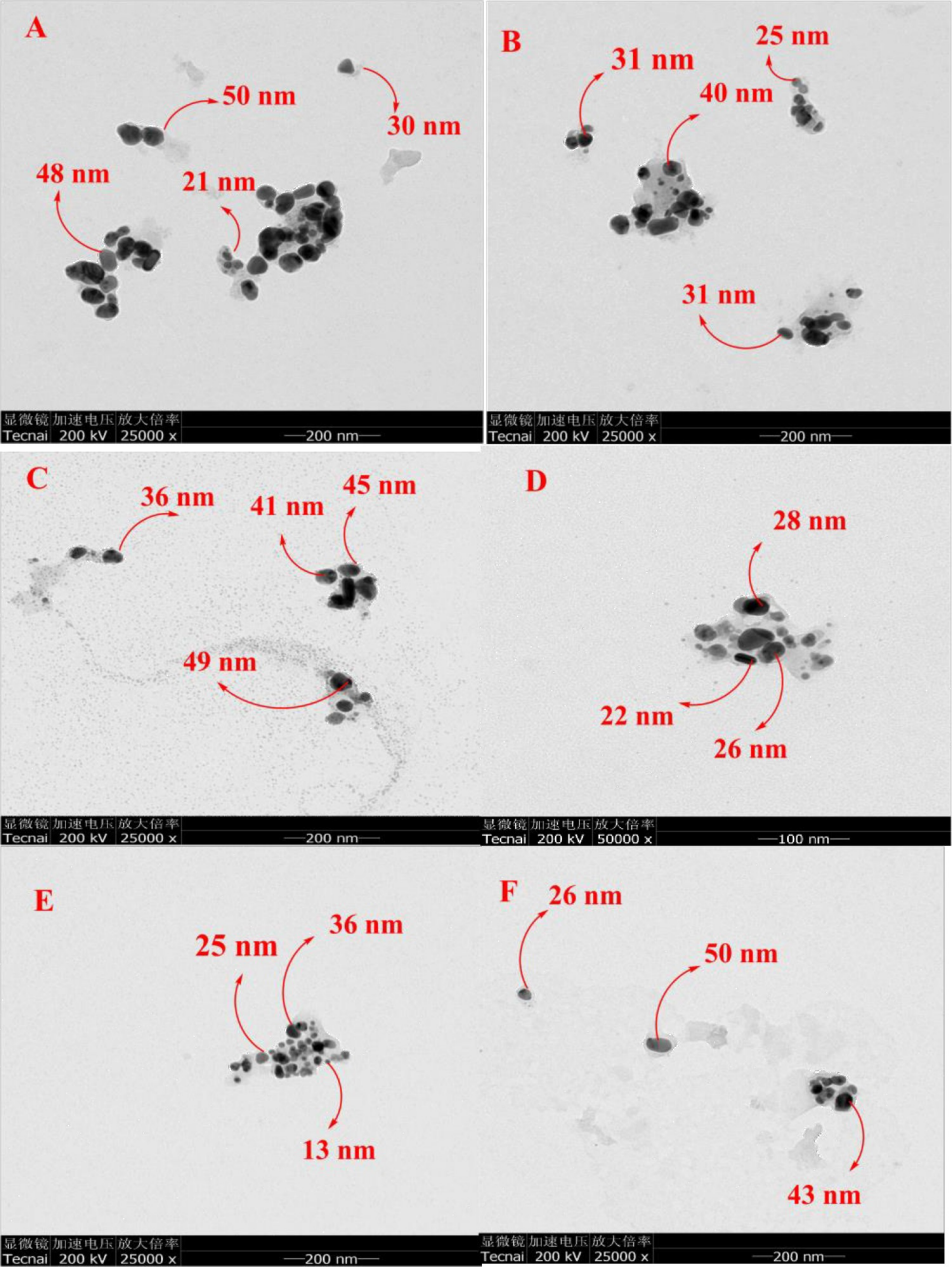
Transmission electron microscopy (TEM) analysis of R-AgNPs **(A, B)**, S-AgNPs **(C, D)** and RS-AgNPs **(E, F)**.

TEM was used to characterize the surface morphology, size and shape of biosynthesized AgNPs. Fig 4 represents TEM micrographs documented from the drop coated TEM grid of the AgNPs at optimal condition which displayed spherical shape with the average size of 32 nm, 25.3 nm and 24.6 nm for R-AgNPs, S-AgNPs and RS-AgNPs respectively (Fig 4B, 4D, 4E). TEM images clearly shows that the synthesized AgNPs samples were well dispersed with minimal aggregation. These results are in proximity with the calculated sizes obtained from the XRD analysis. Our results are in accordance with previous reports [43, 44].

### Thermogravimetric analysis (TG/DTA/DTG)

**Fig 5:**
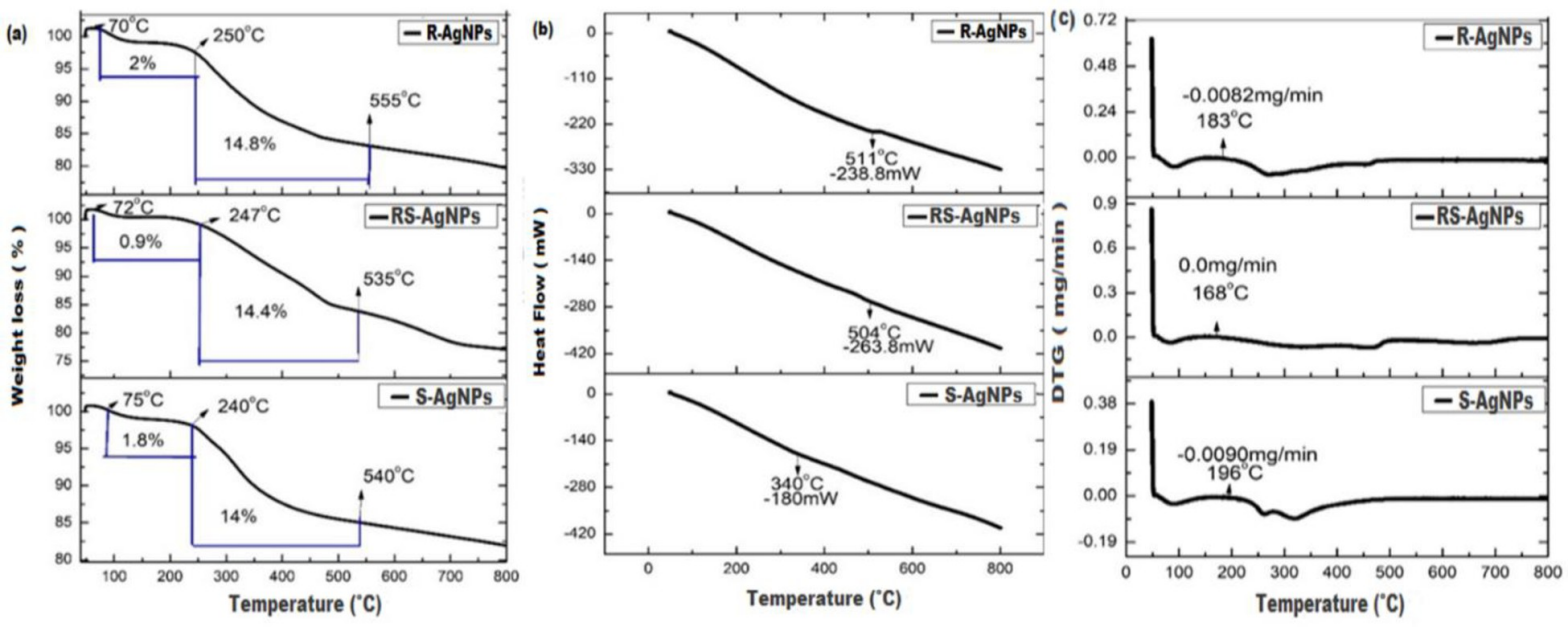
Thermogravimetric (TG) analysis **(a)** Differential scanning calorimetry (DSC) **(b)** and derivative thermogravimetric (DTG) curves **(c)** of R-AgNPs, S-AgNPs and RS-AgNPs.

Thermogravimetric (TG) analysis was performed to find out the percent weight loss of AgNPs with regular increase of temperature (25°C to 800°C). In this study, TGA curve clarifies that the AgNPs percent-weight loss with respect to increase in time was a two-stage process. It was recorded from 75–200°C (S-AgNPs 1.8%, RS-AgNPs 0.9% and R-AgNPs 2%) and 240–550°C (S-AgNPs 14%, RS-AgNPs 14.4% and R-AgNPs 14.8%). Significant percent weight loss of all AgNPs was observed from 500°C to 600°C and almost no change was observed below 70°C and above 700°C (Fig 5A), which can be accredited to the evaporation of water molecules and some organic components. Overall, a high percent weight loss of the R-AgNPs (16.8%) followed by S-AgNPs (15.8%) and RS-AgNPs (15.3%) was recorded. According to the differential scanning calorimetry (DSC) curve, the S-AgNPs, RS-AgNPs and R-AgNPs showed endothermic peaks at 340°C, 504°C and 51°C, respectively (Fig 5B). The denaturation enthalpy of AgNPs was in the range of second stage of compound decomposition, as obtained from the curve of TGA. The major percent weight loss for AgNPs in the temperature range 240°C to 550°C showed a linear relation with thermogravimetric study and denaturation temperature by DSC curve. In the DTG curve (Fig 5C), the peaks at 183°C, 168°C and 196°C by R-AgNPs, RS-AgNPs and R-AgNPs indicated exothermic reactions from which it could be concluded that loss in weight is due to decomposition of organic residues. Similar work showing the AgNPs TGA analysis was also reported previously [45].

### Antioxidant studies

A variety of factors including different diseases causes oxidative stress. Oxidative stress is caused by reactive oxygen species (ROS) which can be extremely dangerous for living organisms as it can result in cellular and DNA damage. Antioxidants are natural mitigating agents of ROS and hence an important weapons against oxidative stress. Recently, green synthesized NPs are being tested for their antioxidant potential. In vitro antioxidant activity was performed using DPPH as free radical. At different concentrations i.e. 400–100 μg/mL, the mean of highest percent radical scavenging was detected in smaples of S-AgNPs (77 ± 1.5) followed by R-AgNPs (71 ± 1.3) and RS-AgNPs (69 ± 1.4), with IC50 value less than 100 μg/ml. Total antioxidant capacity specifies the capability of a compound to reduce reactive oxygen species (ROS). In this activity, a greenish Mo(V)-phosphate complex (Max. absorption at 695 nm) develops from the renovation of Mo(VI) to Mo(V). Relations of three AgNPs samples with ascorbic acid equivalents/mg were recorded as 122 ± 0.69 for the RS-AgNPs followed by the R-AgNPs (120 ± 1.1) and S-AgNPs (61 ± 1.1) at sample concentration of 4 mg/ml. Similarly, assay was performed for the determination of reducing power for the given AgNPs samples at same concentration (4 mg/ml). The value of reducing power was considered in the order of 33 ± 1.2 for RS-AgNPs, 24 ± 0.64 for S-AgNPs and 22 ± 1.2 for the R-AgNPs. The results of antioxidant potential of S-AgNPs, R-AgNPs and RS-AgNPs have been summarized in Table 1. Previous studies have shown the antioxidant activities of bioinspired AgNPs synthesized from root extract of Nepeta leucophylla, and aerial extract of Lippia nodiflora respectively [46, 47]. [48] also reported a dose dependent antioxidant potential for C_60_-AgNPs Nanocomposites. Such promising antioxidant potential of green synthesized NPs validates their importance as novel tools against plethora of diseases.

### Protein kinase assay

The enzyme protein kinase play critical role in almost every cellular function including metabolism, division, proliferation and survival. It also plays key role in the hypae formation of the Streptomyces strain 85E [49]. Studying protein kinase inhibition can pave way for effective treatment of multiple diseases including cancer and overcoming microbial resistance. In this study, AgNPs were evaluated for their inhibition potential of protein kinases. Disc diffusion assays revealed significant inhibition zones (13–7 mm) by 4 mg/ml to 0.5 mg/ml of R-AgNPs, S-AgNPs and RS-AgNPs (Table 2). A dose dependent protein kinase inhibition was observed for all the tested AgNPs. The results showed significant inhibition value when exposed to R-AgNPs as compared to the other tested NPs. At maximum concentration (4 mg/ml), the inhibitory potential of R-AgNPs and S-AgNPs was almost similar that of control. Previously, Ahmed et al. (2014) [50] have studied the protein kinase activity of green synthesized AgNPs synthesized via grape and tomato juices and reported significant results (15–10 mm zone of inhibitions) for concentration of 5 mg/ml. Effective inhibition of protein kinase when exposed to AgNPs means that these NPs would prove to be a weapon of choice against cancer however, further investigations, both in-vitro and in-vivo must be conducted.

**Table 2:** Protein kinase inhibition potential of R-AgNPs, S-AgNPs and RS-AgNPs using Streptomyces 85E strain.

S.No.	Samples	Treatment concentrations (mg/ml)				
		4	2	1	0.5	Control
		Zone of inhibition (mm)				
1	R-AgNPs	13 ± 0.9	12 ± 0.6	12 ± 0.5	11 ± 0.7	15
2	S-AgNPs	13 ± 0.7	1 3± 0.7	12 ± 1.1	7 ± 1	14.6
3	RS-AgNPs	12 ± 1	11 ± 1.1	10 ± 0.6	8 ± 1.1	16

### α-amylase inhibition

**Fig 6:**
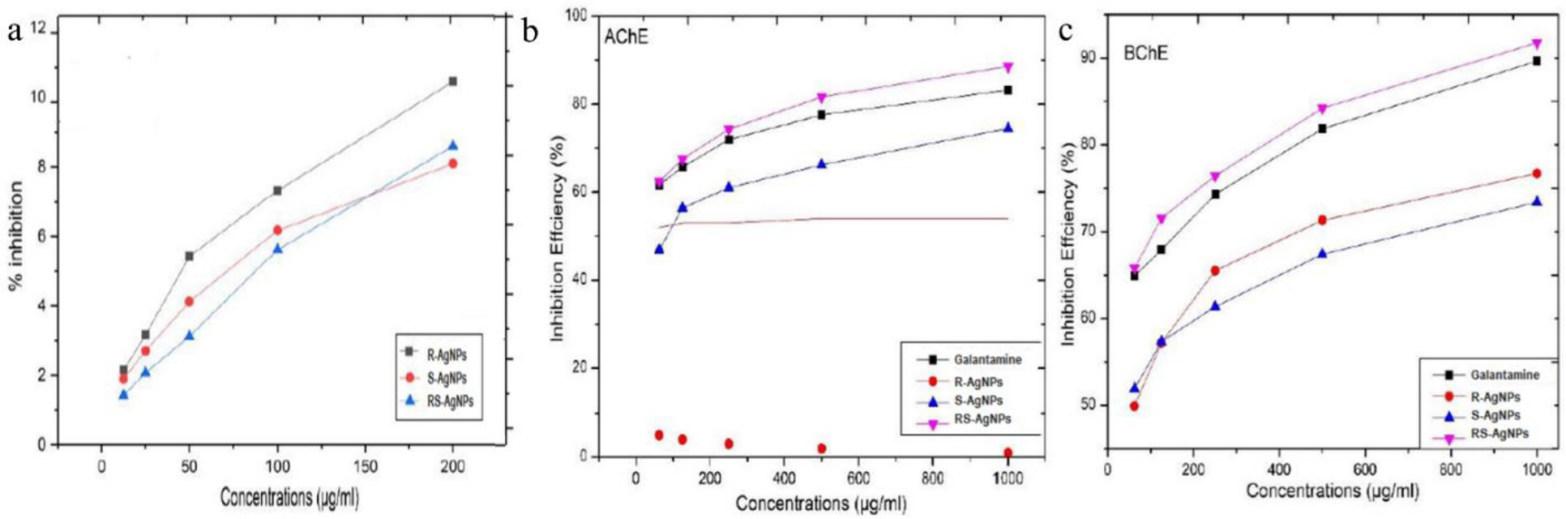
Percent inhibition of α-amylase **(a)**, acetylcholinesterase (AChE) **(b)** and butyryl-cholinesterase (BChE) **(c)** of R-AgNPs, S-AgNPs and RS-AgNPs.

An effective strategy to keep adequate glucose level in blood is the inhibition of carbohydrate hydrolyzing enzymes within acceptable range [51]. The enzyme α-amylase is responsible for the breakdown of carbohydrates into glucose which increases the blood glucose level. Effective inhibition of α-amylase may play critical role in maintaining blood glucose level. In this study, the antidiabetic potency of AgNPs was evaluated at a concentration of 200 μg/ml in order to assess α-amylase percent inhibition when subjected to AgNPs (Fig 6A). The R-AgNPs showed maximum inhibition potential of 10.45%, followed by RS-AgNPs of 8.6%. The variation in α-amylase inhibition potential may be attributed to morphological and physicochemical features resulted from various capping and reducing agent in the extracts. The phytochemicals present in plant extract that were responsible for capping and stabilization of AgNPs and the size of AgNPs may have played vital role in the inhibition of α-amylase.

### Anti-cholinesterase inhibition

In this study, various concentrations of AgNPs (1000 μg/ml to 62.5 μg/ml) used for the inhibition of acetylcholinesterase (AChE) and butyryl-cholinesterase (BChE). The AgNPs exhibited concentration-dependent patterns i.e. greater enzyme inhibition was observed as the AgNPs concentration was raised (Fig 6B, 6C). The RS-AgNPs showed higher inhibitory effect (62.44 ± 0.58% for AChE and 65.80 ± 1.50% for BChE) at lowest concentrations of 62.5 μg/mL, as compared to the same dose of positive control (Galantamine). Furthermore, the inhibition profiles for R-AgNPs and S-AgNPs were 46.9 ± 0.42% (AChE), 51.90 ± 1.16% (BChE) and 46.52 ± 0.38% (AChE), 49.9 ± 0.65% (BChE), respectively. Their calculated IC50 values were noted as 27.5 μg/ml (AChE) and 22.60 μg/ml (BChE), 78.12 μg/ml (AChE) and 61.50 μg/ml (BChE), 91.07 μg/ml (AChE) and 52.35 μg/ml (BChE) for RS-AgNPs, R-AgNPs and S-AgNPs respectively. From the given results, it is concluded that the inhibitory potential of the AgNPs in AChE was higher than the BChE. Wang et al. [52] proposed that the NPs inhibitory effect is primarily produced by interacting with AChE protein. Until now, it has not been explored that in what manner these NPs interact with ChEs proteins. NPs have binding affinity to ChEs owing to the NPs lipophilicity and the hydrophobicity. Rajakumar et al. [53] performed the cholinesterase activity of the AgNPs synthesized from the flower extract of Millettia pinnata and reported that NPs presented remarkable increase in activity toward ChE than plant extract.

### Antimicrobial assays

**Fig 7:**
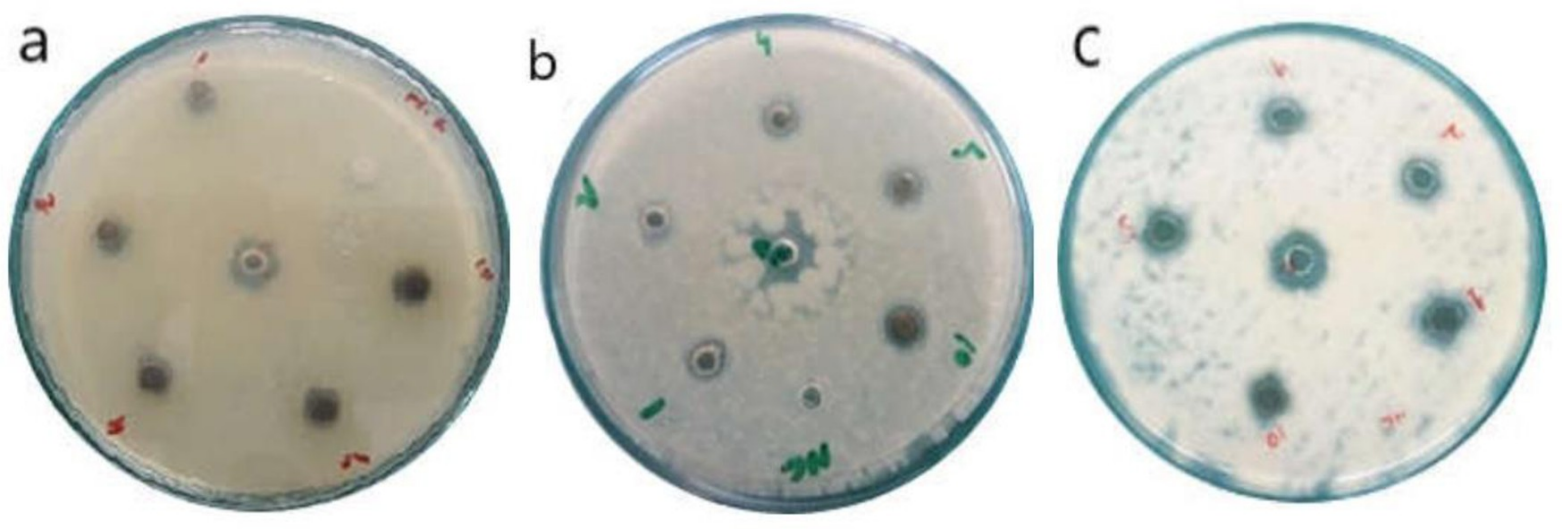
Representative images of antimicrobial activity of synthesized AgNPs against *Staphylococcus epidermidis* (a), *Pseudomonas aeruginosa* (b) and *Fusarium solani* (c).

The antibacterial and antifungal activities of the synthesized AgNPs were assessed by measurement of the diameter of their zones of inhibition and compared with those of positive controls. In antibacterial assays, the highest tested concentration of synthesized AgNPs (10 mg/ml) exhibited maximum inhibition zones against *S*. *epidermidis*, *B*. *subtilis*, *P*. *aeruginosa* and *K*. *pneumonia* (Table 3, Fig 7). Further, the RS-AgNPs were more effective in inhibiting the growth of all tested bacterial strains compared to R-AgNPs and S-AgNPs. The lowest concentration of RS-AgNPs (1 mg/ml) and positive control (Penicillin) showed comparable zones of inhibition against S. epidermidis. Overall, the antibacterial trend exhibited by AgNPs was found to be dose-dependent, as evident by previous studies [54]. However, lower concentrations of some NPs were more effective against bacterial strains than higher concentrations. For example, 1 mg/ml of S-AgNPs exhibited higher zones of inhibition against *P*. *aeruginosa* and *K*. *pneumonia* than its higher concentrations. Similarly, 1 mg/ml of S-AgNPs was more effective against *B*. *subtilis* than 10 mg/ml. This may be attributed to the agglomeration-free NPs in lower concentrations that could be easily taken up by bacterial cells. In previous studies, moderate antibacterial activities were reported for AgNPs synthesized from *Givotia moluccana* leaf extract and root extract of *Diospyros sylvatica* [43, 44]. Such antimicrobial potential of AgNPs can be attributed to structural changes in the cell membrane of the microbes. Additionally, the AgNPs may cause damage to the enzymes and DNA present within the microbes which ultimately results in cellular death [55].

**Table 3:** Results of the antibacterial potentials of R-AgNPs, S-AgNPs and RS-AgNPs against pathogenic strains.

			Treatment Concentrations (mg/ml)					
S.No.	Organisms	Samples	10	5	4	2	1	Penicillin (1.0 mg/ml)
			Zone of Inhibition (mm)					
1	*S*. *epidermidis*	R-AgNPs	8±0.98	7.5±0.78	7±0.91	6.5±0.78	6±0.09	11±1.09
		S-AgNPs	9±0.78	8.5±0.84	7.5±0.79	7±0.69	7±0.59	12±1.12
		RS-AgNPs	18.3±2.05	15.9±1.14	14.1±2.01	13.2±1.41	11.4±0.91	21.9±2.31
2	*B*. *subtilis*	R-AgNPs	16.5±1.89	13.9±1.51	12.8±1.78	11.1±1.09	9.8±1.12	30±2.98
		S-AgNPs	7.2±0.67	8±0.89	7.1±0.97	8.6±0.99	6.5±0.98	23.7±2.23
		RS-AgNPs	19.7±1.59	15±1.09	13.6±1.51	12.7±1.09	12.1±1.09	35±3.01
3	*P*. *aeruginosa*	R-AgNPs	6.5±0.78	6.5±0.76	6.6±0.98	6±0.90	5.6±0.97	18.2±2.98
		S-AgNPs	7±0.69	8.6±0.89	8.2±0.98	6.7±0.93	7.7±0.89	14.2±1.29
		RS-AgNPs	8.2±0.99	7.2±0.79	7.2±0.97	7±0.95	7.5±0.89	21.5±2.09
4	*K*. *pneumoniae*	R-AgNPs	11.2±1.13	11.1±1.04	9.9±0.99	7.2±0.95	6.1±0.99	13.5±1.98
		S-AgNPs	7.7±0.89	7.9±0.69	7.8±0.89	7.2±1.08	8.8±0.90	17±2.03
		RS-AgNPs	15.2±1.11	15±1.13	14.9±2.10	13.5±1.98	12.7±1.09	20±2.00
5	*F*. *solani*	R-AgNPs	11.5±1.21	10.4±0.99	10.1±1.12	9.9±1.01	8.5±0.89	8±0.99
		S-AgNPs	11.8±1.07	8.5±0.91	7.8±0.89	6.6±0.69	6.2±1.01	6±0.87
		RS-AgNPs	11±1.21	10.5±1.08	9.4±0.89	9.1±1.09	8.7±0.98	7±0.67
6	*A*. *flavus*	R-AgNPs	12.2±1.43	10.3±1.11	10±0.79	9.1±0.89	8.2±1.21	8±0.99
		S-AgNPs	13.2±1.23	10.4±1.05	10.9±1.40	11.4±1.79	10.5±1.34	10.2±1.12
		RS-AgNPs	12±1.09	11.7±0.87	11±1.70	10.5±1.11	10.5±1.12	9.8±0.78
7	*A*. *fumigatus*	R-AgNPs	16.7±1.31	10±0.99	9.1±0.97	7.4±0.89	9.5±0.89	8.2±1.12
		S-AgNPs	11.3±1.09	9±0.98	6.9±0.81	6.5±0.69	6.3±0.99	6.1±0.98
		RS-AgNPs	13.1±1.43	11.5±1.32	10.6±1.07	9.9±1.08	9.4±1.20	9.7±0.92

The antifungal potential of the synthesized AgNPs was assessed against *A*. *flavus*, *A*. *fumigatus* and *R*. *solanai* strains of fungi according to their zone of inhibition in comparison with the activity of the standard, amphotericin B. AgNPs were found effective against all the fungal strains studied. At lowest concentration of 1 mg/ml, S-AgNPs showed highest zones of inhibition against *A*. *flavus* (10.2 mm), followed by RS-AgNPs against *A*. *flavus* and *A*. *fumigatus* (9.8 mm and 9.7 mm) respectively (Table 3). R-AgNPs presented lowest inhibition zone with diameter ranging between 8–8.2 mm against all the 3 pathogenic fungal strains at lowest concentration (1 mg/ml). The zones of inhibition increased gradually as the concentrations of samples increases from 1–10 mg/ml. The results of all three-tested samples were close to the mean value of positive control (12.5 mm). The antifungal activities of biosynthesized silver AgNPs against the same fungal species have previously been reported. AgNPs synthesized using *A*. *niger* effectively inhibited the growth of *A*. *flavus* and *A*. *fumigatus* (showing 13 ± 2 and 14 ± 2 mm zone of inhibition respectively). Similarly, AgNPs synthesized using aqueous extract of *Plumbago capensis* also showed excellent antifungal potential against *A*. *fumigatus* [45, 46]. In general, the antimicrobial potential of AgNPs is attributed to the size and shape of the NPs. NPs due to their miniscule size can easily penetrate microbial cell wall which may cause cell wall disruption. NPs upon entry to the cell may also inhibit enzymes and damage the DNA which is chiefly responsible for the antimicrobial effect of the NPs [56].

### Cytotoxic activities

#### Human Red Blood Cells (hRBCs) compatibility test

**Fig 8:**
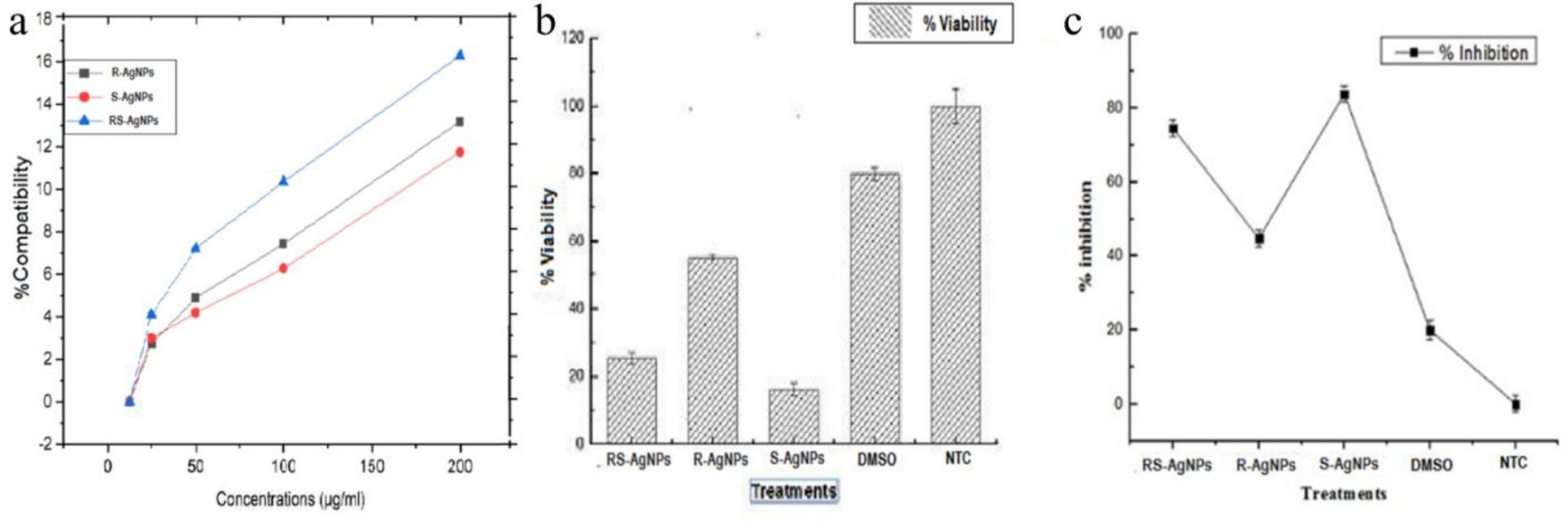
RBCs hemolytic assay **(a)** percentage viability **(b)** and percent inhibition **(c)** of cells relative to untreated control (Mean ± SD).

In this study, different concentrations of the synthesized AgNPs evaluated for their hemolysis of RBCs, and the subsequent release of hemoglobin, showed no hemolysis (Fig 8A). According to “American Society for “Testing and Materials Designation” those substance or materials causing hemolysis > 5%, 2–5% and < 2% are said to be hemolytic, slightly hemolytic and non-hemolytic materials, respectively [47]. The observed non-hemolytic profile of these AgNPs could be attributed to their greener origin, their biocompatible surface chemistry, size, and physiochemical properties [57]. It has been reported via various studies that AgNPs possess lesser hemolytic properties than their Ag+ ions constituents [58]. This is then supplemented with the fact these AgNPs are synthesized via plant-based greener routes. Plant secondary metabolites capping these Ag ions to fabricate AgNPs might have conferred them the ability to kill pathogenic bacteria and at the same time be compatible with biological system containing blood cells.

#### Cytotoxicity of NPs against HepG2 cell line

**Fig 9:**
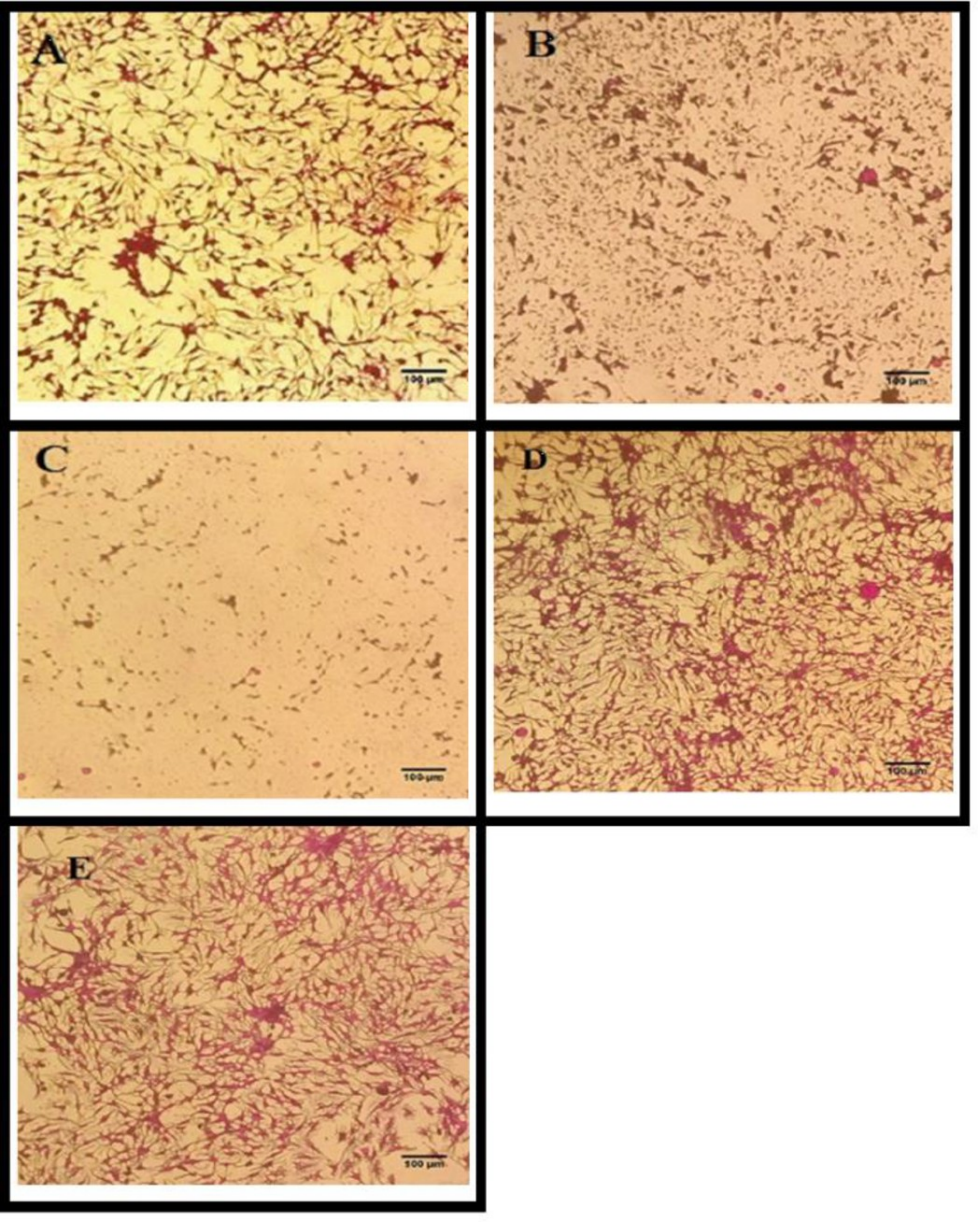
Cytotoxicity of synthesized AgNPs against cancerous HepG2 cell lines upon treatment for 24 hrs. Magnification = 200X, Scale = 100 μm. **A** = R-AgNPs, **B** = S-AgNPs, **C** = RS-AgNPs, **D** = untreated cells, **E** = DMSO 1% (negative control).

In vitro cytotoxicity of the AgNPs was screened using HepG2 cell line through SDB assay. The cytotoxic effects of AgNPs have proved them to be the center of contemplation when it comes to anti-cancer therapies. AgNPs have the potential to disrupt mitochondrial respiratory chain. This happens due to the production of ROS induced by AgNPs which inhibits ATP synthesis and subsequently damaging the DNA [59]. Oxidative stress caused by NPs have been reported to induces mitochondrial dysfunction which suppress the division of cancerous cells [60] In this assay, cells were treated with the synthesized AgNPs (200 μg/ml) for 24hrs. In cytotoxicity of HepG2 cell lines by AgNPs (200 μg/ml) exhibited strong activity by S-AgNPs (83.82%), followed by RS-AgNPs (74.55%) and R-AgNPs (44.82%) (Fig 8B, 8C). Some morphological modifications in cancerous cells were also observed in response to AgNPs treatment. Small round to fibrous type cells verified the impact of inhibition (Fig 9). A similar study of the Hep-G2 cell viability was reported previously [61]. The cytotoxicity of AgNPs is a direct result of oxidative stress induced by Ag ion release from AgNPs. This oxidative stress is achieved by lowering superoxide dismutase (SOD) and glutathione (GSH) levels and promoting lipid peroxidation, which triggers apoptosis by increasing caspase-3 activity and DNA fragmentation [62]. AgNPs interact with thiol groups in reduced GSH and proteins, such as thioredoxin, SOD, and thioredoxin peroxidase. It has also been shown that the cytotoxicity of AgNPs depends on time, dose, and temperature in which the AgNPs are applied, their size, surface properties and cell type. AgNP exposure may cause changes/reduction in cell shape, viability of cells, and then increase in the enzyme lactate dehydrogenase (LDH) which ultimately cause cell death and necrosis [63]. For instance, the morphology of epithelial cells can change when significant concentrations of AgNPs are present, becoming less polyhedral and more fusiform, shrunken, and rounded. Internalized AgNPs can also damage cell membrane integrity, produce lysosomal swelling, and even rupture lysoso-mal membranes. Thiol groups in decreased GSH and proteins like thioredoxin, SOD, and thioredoxin peroxidase are preferred by AgNPs and released Ag ions [64].

## Conclusion

Green biotechnology is an innovative and growing resource in the search for more environmentally benign processes. Metallic NPs are most widely applicable in the field of medical sciences, therefore; our study involved biosynthesis of AgNPs from medicinal plant *D*. *Uncinatum*, and to test the synthesized NPs for their cytotoxic potential against cancer cells, protein kinase, alpha amylase, anti-cholinesterase, anti-microbial, antioxidants activities and to evaluate their phytochemical analysis. We found strong cytotoxic effect against cancer cell lines by hexagonal S-AgNPs. The highest antioxidant, anti-cholinesterase and antibacterial activities were shown by RS-AgNPs and the highest antifungal activity was shown by S-AgNPs. Similarly, R-AgNPs showed maximum inhibition of alpha amylase and protein kinase. RS-AgNPs showed maximum phenolics and flavonoids, suggesting their positive correlation with antioxidant and antibacterial activities. Furthermore, the cytotoxic and antifungal activities of S-AgNPs may be attributed to the synergism of NPs and secondary metabolites other than phenolics and flavonoids. NPs size play a critical role in their biological potential. Owing to their small size, NPs can easily penetrate into the cell causing oxidative stress. Apart from this, NPs also results in cell wall and DNA damages which causes cell death. This feature of NPs makes it suitable for its use as antibacterial and antifungal agent and can be a future target for coping with rising issue of antimicrobial resistance. The synthesized AgNPs also exhibited cytotoxic potential and resulted in inhibition of protein kinase which is promising indicator for cancer therapies in future.

## Supporting information

S1 Graphical abstract Graphical abstract of the research work. (TIF)
