# Supplementary figures and images for: Delpinium uncinatum mediated green synthesis of AgNPs and its antioxidant, enzyme inhibitory, cytotoxic and antimicrobial potentials
Source: PLoS One. 2023 Apr 4;18(4):e0280553. doi: 10.1371/journal.pone.0280553 (PMC10072477; doi:10.1371/journal.pone.0280553)

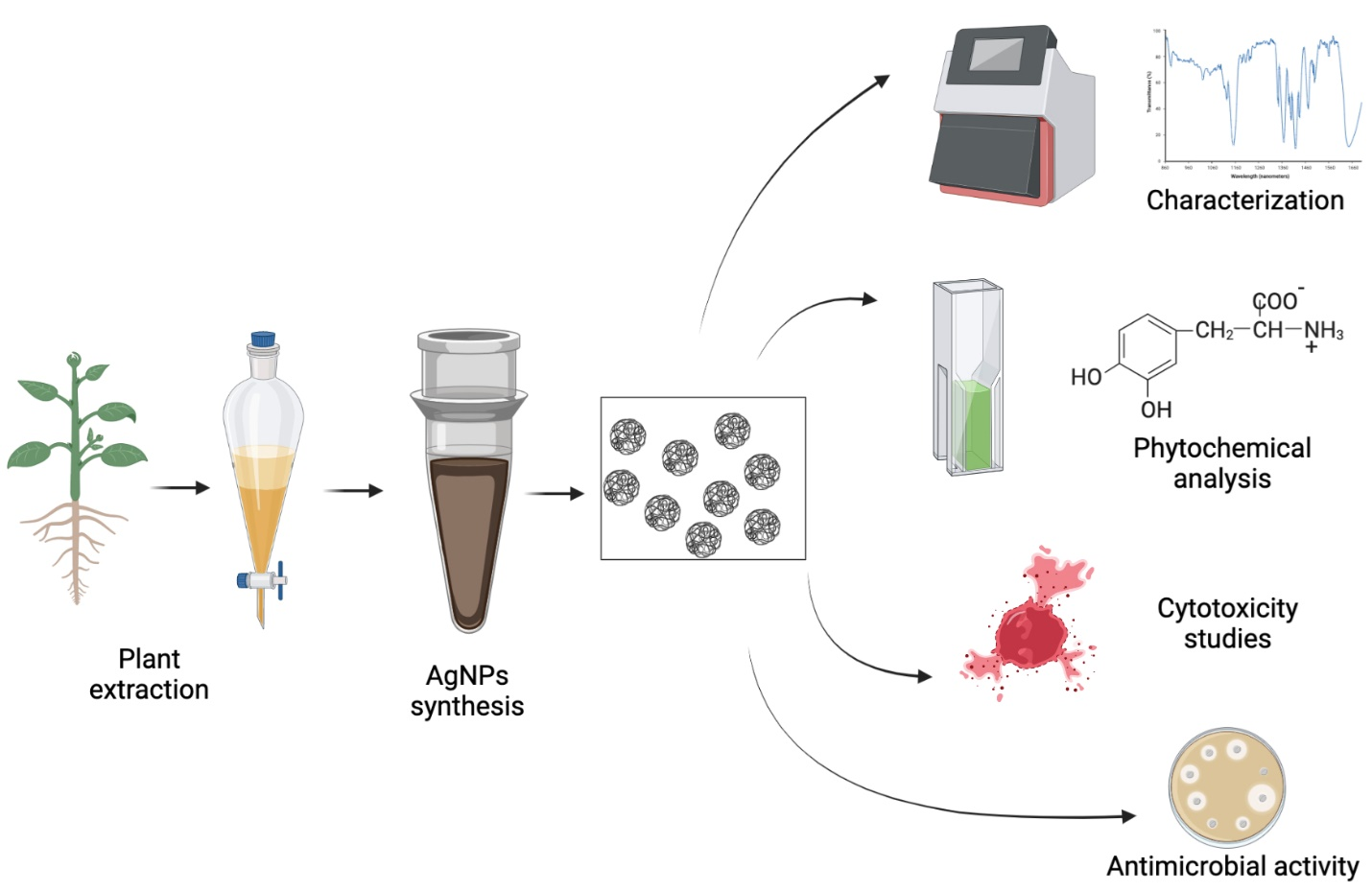

Supplement: S1 Graphical abstract — (TIF) [file pone.0280553.s001.tif]
